# Distinctive Archaeal Composition of an Artisanal Crystallizer Pond and Functional Insights Into Salt-Saturated Hypersaline Environment Adaptation

**DOI:** 10.3389/fmicb.2018.01800

**Published:** 2018-08-14

**Authors:** Alvaro M. Plominsky, Carlos Henríquez-Castillo, Nathalie Delherbe, Sheila Podell, Salvador Ramirez-Flandes, Juan A. Ugalde, Juan F. Santibañez, Ger van den Engh, Kurt Hanselmann, Osvaldo Ulloa, Rodrigo De la Iglesia, Eric E. Allen, Nicole Trefault

**Affiliations:** ^1^Department of Oceanography, Faculty of Natural and Oceanographic Sciences, Universidad de Concepción, Concepción, Chile; ^2^Instituto Milenio de Oceanografía, Concepción, Chile; ^3^Biology Department, Cell and Molecular Biology Joint Doctoral Program with UC San Diego, San Diego State University, San Diego, CA, United States; ^4^Marine Biology Research Division, Scripps Institution of Oceanography, University of California, San Diego, La Jolla, CA, United States; ^5^Programa de Doctorado en Ingeniería de Sistemas Complejos, Universidad Adolfo Ibáñez, Santiago, Chile; ^6^uBiome, Inc., San Francisco, CA, United States; ^7^Center for Bioinformatics and Integrative Biology, Facultad de Ciencias Biológicas, Universidad Andrés Bello, Santiago, Chile; ^8^Center for Marine Cytometry, Concrete, WA, United States; ^9^Department of Earth Sciences, ETH Zürich, Zurich, Switzerland; ^10^Department of Molecular Genetics and Microbiology, Faculty of Biological Sciences, Pontificia Universidad Católica de Chile, Santiago, Chile; ^11^Division of Biological Sciences, University of California, San Diego, La Jolla, CA, United States; ^12^GEMA Center for Genomics, Ecology and Environment, Faculty of Sciences, Universidad Mayor, Santiago, Chile

**Keywords:** hypersaline environments, solar salterns, metagenomics, microbial ecology, environmental adaptation, functional metagenomics, artisanal crystallizer pond

## Abstract

Hypersaline environments represent some of the most challenging settings for life on Earth. Extremely halophilic microorganisms have been selected to colonize and thrive in these extreme environments by virtue of a broad spectrum of adaptations to counter high salinity and osmotic stress. Although there is substantial data on microbial taxonomic diversity in these challenging ecosystems and their primary osmoadaptation mechanisms, less is known about how hypersaline environments shape the genomes of microbial inhabitants at the functional level. In this study, we analyzed the microbial communities in five ponds along the discontinuous salinity gradient from brackish to salt-saturated environments and sequenced the metagenome of the salt (halite) precipitation pond in the artisanal Cáhuil Solar Saltern system. We combined field measurements with spectrophotometric pigment analysis and flow cytometry to characterize the microbial ecology of the pond ecosystems, including primary producers and applied metagenomic sequencing for analysis of archaeal and bacterial taxonomic diversity of the salt crystallizer harvest pond. Comparative metagenomic analysis of the Cáhuil salt crystallizer pond against microbial communities from other salt-saturated aquatic environments revealed a dominance of the archaeal genus *Halorubrum* and showed an unexpectedly low abundance of *Haloquadratum* in the Cáhuil system. Functional comparison of 26 hypersaline microbial metagenomes revealed a high proportion of sequences associated with nucleotide excision repair, helicases, replication and restriction-methylation systems in all of them. Moreover, we found distinctive functional signatures between the microbial communities from salt-saturated (>30% [w/v] total salinity) compared to sub-saturated hypersaline environments mainly due to a higher representation of sequences related to replication, recombination and DNA repair in the former. The current study expands our understanding of the diversity and distribution of halophilic microbial populations inhabiting salt-saturated habitats and the functional attributes that sustain them.

## Introduction

Solar salterns provide tractable model ecosystems for understanding microbial selection, successions, and habitat resilience, and are thus one of the most studied hypersaline environments. These are engineered pond systems for the commercial production of halite (NaCl) that can be found in many regions throughout the world, particularly in tropical and subtropical areas (Oren, 2002, 2013a; Ventosa et al., 2014). As the water passes through a series of evaporation ponds, it generates a discontinuous gradient of increasing salinities, beginning close to that of the surrounding input water and ending at the crystallizer pond, where salinities reach the crystallization point of halite (Oren, 2002, 2013a; Ventosa et al., 2014).

The extreme osmotic stress associated with hypersaline habitats elicits strong selective pressures for adaptations that sustain life. Cellular life in hypersaline habitats is dominated by halophilic archaea and bacteria, with a few microbial eukaryotes, such as photosynthetic microalgae, heterotrophic protists and fungi, and the crustacean *Artemia salina* (Ventosa et al., 2014). Microbial inhabitants of hypersaline environments have developed specialized adaptations to live under the high ionic strength of these systems (Oren, 2002, 2013a,b). “Low-salt-in” halophilic microorganisms maintain lower intracellular salt concentrations than that of the external environment (especially Na^+^). In contrast, “high-salt-in” halophiles accumulate high concentrations of inorganic ions in the cytoplasm, usually K^+^, and Cl^-^. While “high-salt-in” halophiles are physiologically constrained to environments with a constant presence of high salt concentrations, “low-salt-in” halophiles can regulate the intracellular concentrations of their compatible solutes accordingly to approximate the prevailing environmental salinity (Ventosa et al., 1998; Oren, 2013a). Genomic and structural analysis of halophilic Archaea and many halophilic Bacteria indicate that these microorganisms have shifted toward acidic proteomes to enable the correct folding and performance of their proteins under such conditions (DasSarma and DasSarma, 2015).

Metagenomic analyses have been conducted in several hypersaline environments across a broad range of salt concentrations. These studies have shown that the community structure exhibited by salt-saturating hypersaline environments, for example, late-stage crystallizer ponds with salinities >30%, generally present a high dominance of Archaea, such as *Haloquadratum walsbyi* (phylum Euryarchaeota), and Bacteria, such as *Salinibacter ruber* (phylum Bacteroidetes) (Oren, 2002, 2013a). In contrast, sub-saturated hypersaline environments present a greater diversity of halophilic representatives from diverse phyla, including Proteobacteria, Firmicutes, Cyanobacteria, Bacteroidetes, Spirochaetes, and methanogenic Euryarchaeota (Ventosa et al., 2015).

The Cáhuil Solar Saltern consists of a series of artisanal ponds at the shores of the Cáhuil Lagoon that forms at the mouth of the Nilahue creek, in central Chile. During the summer, it is separated from the ocean by a sandbar that forms due to a decrease in the water flux from the creek and by high littoral sediment transport from the coast. During winter, the higher water flux from the creek transforms the Cáhuil Lagoon into a seasonally stratified estuary by breaking through the sandbar and connecting it with the South Pacific Ocean. This seasonal dynamic drastically transforms the hydrological properties of the Cáhuil Lagoon with regards to its surface temperature (22.1°C in summer and 10.5°C in winter) and salinity (ranging from 2.4 to 2.2% [w/v] in summer and from 3.1 to 0.01% in winter, in locations nearest to the sea and the creek, respectively) (Andrade and Grau, 2005).

This study describes the physico-chemical properties and microbial communities inhabiting the Cáhuil Solar Saltern ponds. Comparative metagenomic analysis of the Cáhuil microbial ecosystem against 25 other hypersaline metagenomes collected worldwide provides insight into the abiotic-biotic coupling, functional convergence, and the distinctive metabolic adaptations that unite or distinguish the microbial communities that define extreme hypersaline habitats.

## Materials and Methods

### Sampling Site and Physical-Chemical Properties

Surface water samples (∼5 L) were collected on January 12, 2012, from five ponds with different salt concentrations at the Cáhuil Solar Salterns (Cáhuil, Chile; 34°30′10” S; 71°59′09” W; **Supplementary Figure 1A**). Temperature and salinity (**Table 1**) were measured on site using a Multi-Parameter Water Quality Monitoring System (U-22XD, HORIBA), and a pH-meter (HI 991001, Hanna). The ion concentrations of Mg^2+^, Ca^2+^, Na^+^, and K^+^ of the Cáhuil salt crystallizer pond were determined by inductively coupled plasma-optical emission spectrometer (ICP-OES; Perkin Elmer Optima 3000DV), as described previously (Janney and Castillo, 1996). Samples were diluted a thousand-fold, until the Na^+^ concentration was at sub-ppm levels, before performing each measurement with 2% ultra-pure (double-distilled) Nitric Acid (HNO_3_^-^). The density of the salt-saturated water sample was determined in a DE45 Delta Range Density Meter to calculate the final concentration of the ions.

**Table 1:** Physical properties, size fraction sampled, and metagenome characteristics for Cáhuil saltern pools, Bras del Port Solar Saltern SS19 and SS37 (Ghai et al., 2011; Fernández et al., 2014), Lake Tyrrell (Narasingarao et al., 2012; Podell et al., 2014) and The Great Salt Lake Rozel Point (Parnell et al., 2010).

Site	Collection date	Salinit*y* (%)	Temperature (°C)	pH	Size fraction sequenced (μm)	Number of reads	Dataset size (Mpb)^1^
Cáhuil saltern CL2.8	12/01/12	2.9	27.5	7.0	-	-	-
Cáhuil saltern CL7.1	12/01/12	7.1	32.0	6.8	-	-	-
Cáhuil saltern CL11	12/01/12	11.3	34.5	6.5	-	-	-
Cáhuil saltern CL28	12/01/12	29.7	37.2	6.5	-	-	-
Cáhuil saltern CL34	12/01/12	34.1	41.2	6.4	3.0 to 0.2	183299	75.13
Santa Pola SS19	21/07/08	19.0	30	8.0	5.0 to 0.2	1072205	348.99
Santa Pola SS37	26/06/08	37.0	41.0	7.1	5.0 to 0.2	656800	230.63
Lake Tyrrell^2^ (HAT/HBT)	03/01/09	29.0	29.5	7.1	3.0 to 0.1	1933848	768.90
Rozel Point^3^ (GSL-RP)	Summer 2007	30.0	26.3	-	Centrifugation^4^	585929	247.42

^*1*^Dataset size after quality filtering of the metagenomic reads (see Materials and Methods). ^*2*^Temperature corresponds to the maximum temperature during collection day. ^*3*^NOAA highest average temperature recorded for Salt Lake City during Summer 2007 (June-August; http://www.wrh.noaa.gov/slc/climate/slcclimate/SLC/pdfs/Seasonal%20Maximum%20and%20Minimum%20Average%20Temperatures.pdf). ^*4*^As described in Parnell et al. (2010).

### Flow Cytometry and Microbial Community Pigment Analysis

Saltern water samples were pre-filtered through a 70 μm nylon mesh before flow cytometry, pigment extraction, and molecular analysis (see below). Samples from each saltern pond were analyzed by flow cytometry using a high speed “jet-in-air” Influx Flow Cytometer (Becton Dickinson, Franklin Lakes, NJ, United States; formerly Cytopeia). The Influx flow cytometer used a combination of 355 nm (Ultra-Violet; UV), 457 nm (Blue), 488 nm (Blue), 532 nm (Green) and 638 nm (Red) excitation lasers and bandpass filters for red (692/40 nm) and orange (579/20 nm) fluorescence. Lasers were aligned and calibrated with 1 μm ultra-rainbow fluorescent beads (Spherotech, Inc., Libertyville, IL, United States). Log amplified signals for the Forward Angle Light Scattering (FALS) plus red signal (i.e., a proxy for chlorophylls content) for the 488, 532, and 638 nm excitation lasers and orange signal (i.e., a proxy for phycobiliproteins and beta-carotene content) for the 457 nm, 488 nm, and 532 nm lasers were recorded. Signal detection was triggered on the FALS. Cytometric analysis of pigment-containing cells was performed using natural (unstained) samples, the same day of their collection. Samples were fixed with paraformaldehyde (final concentration, 1% w/v), and stained with 0.5 μg mL^-1^ of Hoechst 33342 (Monger and Landry, 1993). A 355 nm (UV) laser was used for the detection of Hoechst 33342 stained samples. Emission detected in the blue range was detected using a 460/20 nm bandpass filter.

For each analysis type and sample, 100000 events were recorded using Spigot software (Cytopeia Inc., Seattle, WA, United States) and further processed through the FlowJo software v7.6.1 (Tree Star, Inc.). To test for differences between samples, a non-parametric Wilcoxon rank sum test was applied with a Bonferroni adjustment of the *P*-value.

To extract pigments of the microbial fraction, a 2 mL sample from each saltern pond was centrifuged at 13000 ×*g* for 10 min, and the supernatant was replaced with 100% cold acetone; cells were then re-suspended with a vortex mixer for 30 s, and incubated in the dark at 4°C overnight. Samples were then centrifuged at 13000 ×*g* for 10 min and the clear supernatant was utilized to determine the absorbance of the pigments between 400 and 900 nm with a spectrophotometer (Hach, model DR 2700).

### Microbial Community Processing and Sequencing

For DNA extraction, samples (1 L) were sequentially filtered through 20 μm (Nylon, Millipore), 3 μm (polycarbonate, Millipore) and 0.2 μm pore size filters (polyethersulphone, Millipore). Due to high cell density, only 600 mL were filtered of the salt-crystallizer pond samples. Community DNA was extracted from the 0.2 and 3 μm size filters by the CTAB method (Wilson, 1990). The 0.2 to 3 μm size fraction sample from the crystallizer pond was sequenced at the Center for Genomics and Bioinformatics, Universidad Mayor (Santiago, Chile) using Titanium chemistry through a Roche-454 GS-FLX platform (454 Life Sciences, Bradford, CT, United States). The 222,074 raw reads (average length of 450 bp) of the Cáhuil Solar Saltern crystallizer pond metagenome (hereafter described as CL34) were deposited in the NCBI Sequence Read Archive (SRA) under accession SRX680116 (Plominsky et al., 2014).

### Genetic Fingerprinting and Microbial Community Similarity Analyses

Polymerase chain reaction (PCR) was performed to amplify the genes of SSU rRNA genes for Archaea and Bacteria from the community DNA of each pond. Primers 344f (ACGGGGYGCAGCAGGCGCGA, where ‘Y’ = ‘C’ or ‘T’) and 915r (GTGCTCCCCCGCCAATTCCT) were used for archaeal SSU rRNA amplification (Casamayor et al., 2002). Primers 358f (AGACTCCTACGGGAGGCAGCAGT) (Lane, 1991) and 907r (CCGTCAATTCMTTTRAGTTT, where ‘M’ = ‘A’ or ‘C’ and ‘R’ = ‘A’ or ‘G’) were used for the Bacterial SSU rRNA amplification (Lane et al., 1985). PCR conditions for Archaea and Bacteria SSU rRNA genes consisted of 35 cycles with a touchdown annealing, starting at 71°C and 61°C, respectively (decreasing by 1°C every cycle for the first 10 cycles). Subsequent PCR cycles were performed with annealing temperatures of 61 and 54°C for Archaea and Bacteria, respectively. The PCR products were separated by denaturing gradient gel electrophoresis (DGGE). A 6% (w/v) polyacrylamide gel with a linear DNA-denaturant gradient was generated by mixing solutions of 30–70% (v/v) and 40–80% (v/v) denaturing agent (100% [v/v], consisting of 7 M urea and 40% [v/v] deionized formamide) for Archaea and Bacteria, respectively. DGGE analysis was performed at 60°C and 75 V for 16 h, in a DCode^TM^ system (Bio-Rad). Gels were stained for 45 min with Sybr Gold (1:10000; Molecular Probes), rinsed in Milli-Q water, viewed under UV and photographed. The various bands (**Supplementary Figure 3**) were used to determine the similarities (Bray–Curtis dissimilarity analysis) using single linkage clustering for each sample. All clustering, dissimilarity and similarity profile (SIMPROF) calculations conducted for the fingerprinting and metagenomic analysis (see below) were performed using the Primer 6 software (Primer-E, Plymouth, United Kingdom; Clarke and Gorley, 2006).

### Metagenomic Sequence Assembly and Annotation

The Lake Tyrrell (Victoria, Australia; 35° 19’ 12” S, 142° 48’ 00” E) sequences analyzed here correspond to the HAT and HBT metagenomes collected in January 2009 (hereafter mentioned as HAT/HBT; Podell et al., 2014). The raw reads of HAT/HBT (0.8 to 3 μm and 0.1 to 0.8 μm size fractions, respectively) were uploaded to the NCBI SRA database under BioProject accession number PRJNA388720. The raw reads of the microbial communities of the Bras del Port – Santa Pola Solar Saltern Metagenomes Experiment SS19, SS33, and SS37 (hereafter mentioned as SS19, SS33, and SS37, respectively; Alicante, Spain; 38° 12’ N, 0° 36’ W) (Fernández et al., 2014) were retrieved from the NCBI SRA: Acc. No. SRX090228, SRX347883, and SRX090229, respectively. The Great Salt Lake north arm Rozel Point metagenomic reads (Utah, United States; 41° 25’ 56.13” N, 112° 39’ 48.31” W) (hereafter described as GSL-RP) were downloaded from the JGI Genome projects database^1^ (JGI project ID 400486) (Parnell et al., 2010). Environmental data and sequence statistics of the metagenomes analyzed in this study are available in **Supplementary Data Sheet 2** (“Metagenomes Info”). All raw metagenomic reads were quality filtered for ambiguities and homopolymers (Mothur v1.33.3; Trim.seqs; maxambig = 0; maxhomopol = 10) and clipped to remove low-quality positions (sff_extract 0.3.). Low-quality ends were removed (fastx_trimmer; fastx_toolkit v0.0.13.2.) and reads with <75% of their positions having a Phred quality score ≥ 20 were discarded (fastq_quality_filter; fastx_toolkit v0.0.13.2.). The resulting quality-filtered reads from HAT and HBT were pooled to analyze microbial communities from similar size fractions (i.e., 0.1 to 3 μm). All metagenomic sequence assemblies were performed using Newbler (version 2.7, Roche; parameters: min overlap 80 min overlap identity 99% and seed step 10). The CL34, SS19, SS33, SS37, and GSL-RP assembled contigs and unassembled reads were annotated using the Integrated Microbial Genomes Expert Review pipeline (IMG/ER) (Markowitz et al., 2009), and are publicly available (IMG project IDs 3300000328 [CL34]; 3300006171 [SS19]; 3300006170 [SS33]; 3300001293 [SS37]; 3300000328 and 3300001293 [HAT]; 3300005070 and 3300005075 [HBT]). The GSL-RP metagenome is publicly available through IMG/MER (IMG project ID 2051774008).

### Taxonomy and Functional Similarity of Metagenomes

A Hidden Markov model search for rRNA gene sequences was performed among the quality filtered unassembled metagenomic reads through WebMGA (Wu et al., 2011). This enabled the retrieval of the Archaeal SSU rRNA gene fragments (>400 nucleotides) and those of the LSU rRNA genes of Bacteria and Eukarya (>300 nucleotides). Consensus Archaea SSU rRNA assemblies were built utilizing the “*De Novo* Assembly” tool available in Geneious (v7.1.4; Biomatters^2^,) with the “Highest Sensitivity” method and a 99% sequence identity threshold for the final output sequences. Reference sequences were aligned with MAFFT v7.015b using the E-INS-i algorithm (Katoh and Toh, 2008) and the metagenomic sequences were included utilizing “–add” (Katoh and Frith, 2012). Maximum likelihood phylogenetic inferences were performed with MEGA v5.2 (Tamura et al., 2011). This read-based rRNA gene analysis was complemented with a compositional-based method to determine the taxonomy of a subset of unassembled reads (>100 bp) against the NCBI non-redundant database (v01-2017) using DIAMOND (Buchfink et al., 2015). To have an even dataset to compare against CL34, a subset of 178,049 unassembled reads (>100 bp) were randomly subsampled from SS37, GSL-RP, HAT/HBT, and SS19. The relative abundance of each genus was normalized to its percentages with regards to the total number of reads analyzed. The proportion of each genus was scaled and clustered using the Euclidean distance and ward linkage method. SIMPROF with hierarchical cluster analysis was used to indicate which clusters have non-random structure (Clarke and Gorley, 2006). The significance of the clustering was tested using PERMANOVA. All these analyses were performed in R environment using dedicated functions. The taxonomic composition analysis of the metagenomes included all taxa with >0.9% relative abundance. Results were graphically displayed utilizing Tundra Pie-Chart editor^3^.

The metabolic similarity of 26 saline metagenomes (**Supplementary Data Sheet 2**) was analyzed by determining the relative abundance of 7,733 Clusters of Orthologous Groups (COGs) (Tatusov et al., 2000; Galperin et al., 2014) with categories assigned to at least one of their quality filtered reads (**Supplementary Data Sheet 2**). Of an initial set of 36 metagenomes (**Supplementary Data Sheet 2**), nine were discarded due to having <30,000 quality-filtered reads that could be assigned to the COGs, or because of the possible bias introduced by the multiple displacement amplification procedures performed over their original samples (Yilmaz et al., 2010; Probst et al., 2015). Metagenomic sequences were aligned against the COG protein sequences database using the blastx algorithm of DIAMOND (Buchfink et al., 2015). The result with the highest bitscore was used to assign a COG category to each sequence. Sequences with bitscore values <50 were discarded from further analysis. The relative abundance of each COG category in all hypersaline metagenomes was calculated as [number of quality filtered reads assigned to a COG]/[total number of quality filtered reads of the metagenome that were associated with all COGs]. The top 100 most abundant COGs were utilized to group metagenomes by hierarchical clustering using the Euclidean distances, and Ward method as linkage criteria. PERMANOVA analysis was used to determine the significance of the clustering, and a multiple paired *t*-test was used to determine the COGs that significantly contribute to the clustering, using a *p*-adjusted value <0.05 as cut-off. The *p*-value was adjusted using the Benjamini and Hochberg correction method (Benjamini and Hochberg, 1995). All the analyses were performed in the R environment, using dedicated functions.

## Results and Discussion

### The Cáhuil Solar Saltern Ponds: Physicochemical Conditions and Microbial Community Analysis

The Cáhuil Solar Saltern consists of a series of shallow locks that have been built along the border of the Cáhuil Lagoon to distribute brackish water by passive flow into small ponds and sequentially precipitate carbonate and sulfate salts before the halite crystals are harvested (**Supplementary Figure 1**). This artisanal solar saltern consists of five compartments, from the initial lock to the final crystallizer pond, hereafter described as CL2.8, CL7.1, CL11, CL29, and CL34, based on the corresponding salinity (**Table 1** and **Supplementary Figure 1**). Salinity (S) and temperature (T) of these ponds were measured during sample collection, under an environmental temperature of 24.6°C. S and T increased from the first concentrator pond (CL2.8) to the crystallizer pond (CL34) from 2.85 to 34.1%, and from 27.5 to 41.2°C, respectively (**Table 1**). The pH decreased in the same series of ponds from 7 to 6.4 (**Table 1**).

**FIGURE 1:**
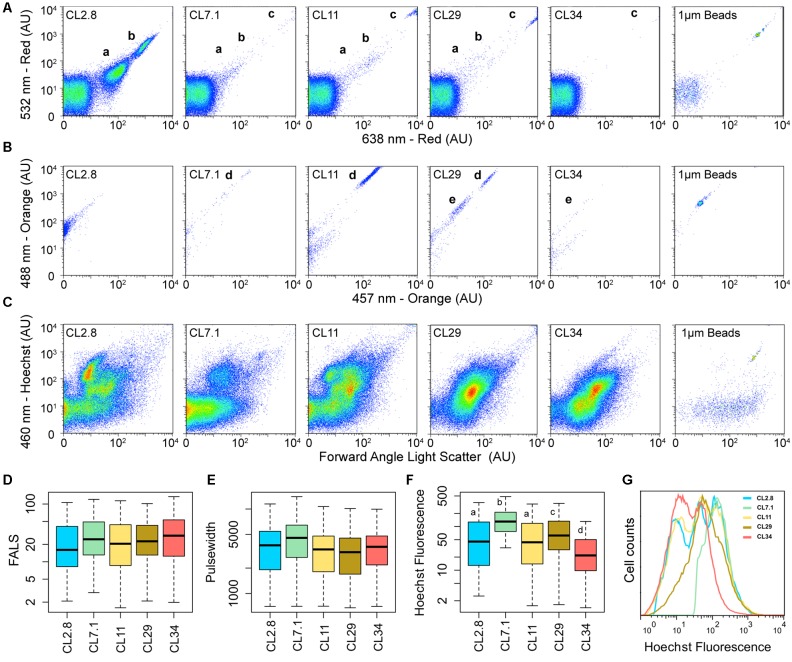
The Cáhuil Solar Saltern microbial communities from ponds CL2.8, CL7.1, CL11, CL29, and CL34 assessed by flow cytometry. **(A)** Red fluorescence, and **(B)** Orange fluorescence. **(C)** Blue fluorescence of Hoechst stained samples and their forward angle light scatter (FALS) after being excited with 355 nm laser. The corresponding fluorescence of 1 μm calibration beads are shown for all cytometric measurements. Boxplots for **(D)** FALS, **(E)** Pulse width and **(F)** Hoechst fluorescence. Lowercase letters denote the statistically significant dissimilarities among the Hoechst fluorescence of microbial communities from different ponds (Wilcoxon rank sum test; *p*-value < 2e^-16^). **(G)** Distribution of Hoechst fluorescence among the microbial communities of the Cáhuil Solar Saltern.

Endogenous pigment properties of microbiota from the Cáhuil Solar Saltern ponds were characterized by flow cytometry. These analyses revealed a transition from an abundance of red-fluorescence positive (a proxy for Chlorophylls)/orange-fluorescence negative cells in pond CL2.8 (groups a and b in **Figure 1A**) to cells with higher red-fluorescence values (group c) and positives for orange-fluorescence (group d) along the increasing salinity gradient up to pond CL29. All Chlorophyll (Chl)*-*containing populations disappeared in CL34. A second group of orange-positive cells (group e) appeared in CL29. Consistent with observed differences in the composition and abundance of their pigment-containing microbial communities, each saltern pond had a distinctive coloration and differed with respect to the optical properties of their pigments (**Supplementary Figure 2**). Ponds CL11 and CL29 had the highest concentrations of Chl (i.e., maximal absorbance at aaa: 440–450 and 660 nm; **Supplementary Figure 2B**), which agreed with the high number of group “c” Chl-containing cells detected by flow cytometry (**Figure 1A**). Also, the pigments extracted from CL29 had two additional major absorbance peaks (at aaa: 480 and 500 nm; **Supplementary Figure 2B**). Phycobiliprotein-containing microorganisms are often considered responsible for emitting orange-fluorescence (by reflecting the red/yellow light of the visible spectrum) (Oi et al., 1982). However, beta-carotenes also reflect this range of the visible light spectrum, with a maximum absorbance for all its isomers within the range of aaa: 400–500 nm (Pfander, 1992). The absorbance profile of CL29 peaks within the Chl range and at aaa: 480 and 500 nm (**Supplementary Figure 2B**) suggesting that the Cáhuil Solar Saltern orange-fluorescence containing groups “e” and “d” likely include beta-carotenes (**Figure 1B**).

Primary production and Chl concentration have been reported to be maximal in saltern ponds with 8–12% salinity, due to the increase of both cyanobacteria and unicellular algae; similar peaks have also been reported in ponds with ∼30% salinity due to the presence of the hypersaline algae *Dunaliella* (Pedrós-Alió, 2004). Since *Dunaliella* spp. are known to synthesize both Chl and beta-carotene (Lers et al., 1991), these peaks most likely correspond to the Chl and beta-carotene-containing cells in Cáhuil Solar Saltern pond CL29.

Changes in the overall microbial community structure along the Cáhuil pond salinity gradient based on DNA composition and FALS properties revealed significant differences in DNA content and composition (Wilcoxon rank sum test; *p*-value < 2e^-16^) (**Figures 1C–G**). The DNA dye Hoechst 33342 used here preferentially stains AT-rich DNA regions. The higher number of events with low signal in CL34 (**Figures 1C,F,G**) is likely due to a higher representation of microorganisms with high-GC content DNA. Many halophilic Euryarchaeota from the family Halobacteriaceae present high-GC genomes (Podell et al., 2014).

The cytometric overview described above was complemented by genetic fingerprinting using the SSU rRNA marker gene. Archaeal SSU genes were detected only in CL11, CL21, and CL34, while Bacteria were amplified in all ponds, with CL34 showing a distinctive bacterial pattern compared to the rest of the ponds (**Supplementary Figure 3**). Subsequently, the microbial composition of the salt-saturated Cáhuil saltern pond CL34 was further assessed by metagenomic sequencing of the 0.2 to 3 μm size fraction.

### Comparative Taxonomic Composition From Metagenomes of Salt-Saturated Aquatic Environments

The taxonomic composition of CL34 was compared to that of other metagenomes from similar environments (**Table 1**). The metagenome from the Bras del Port solar saltern pond SS19 (Ghai et al., 2011) was included as an outgroup from a non-salt-saturated hypersaline environment (i.e., 19% salinity; **Table 1**). All metagenomes analyzed were sequenced by the same technology and collected from similar size fractions during summer seasons except for GSL-RP whose cells were concentrated by centrifugation (Parnell et al., 2010). The uneven sequencing effort from each metagenome (**Table 1**) was accounted for by analyzing the same number of randomly subsampled (quality-filtered) reads from each independent metagenomic data set.

**FIGURE 2:**
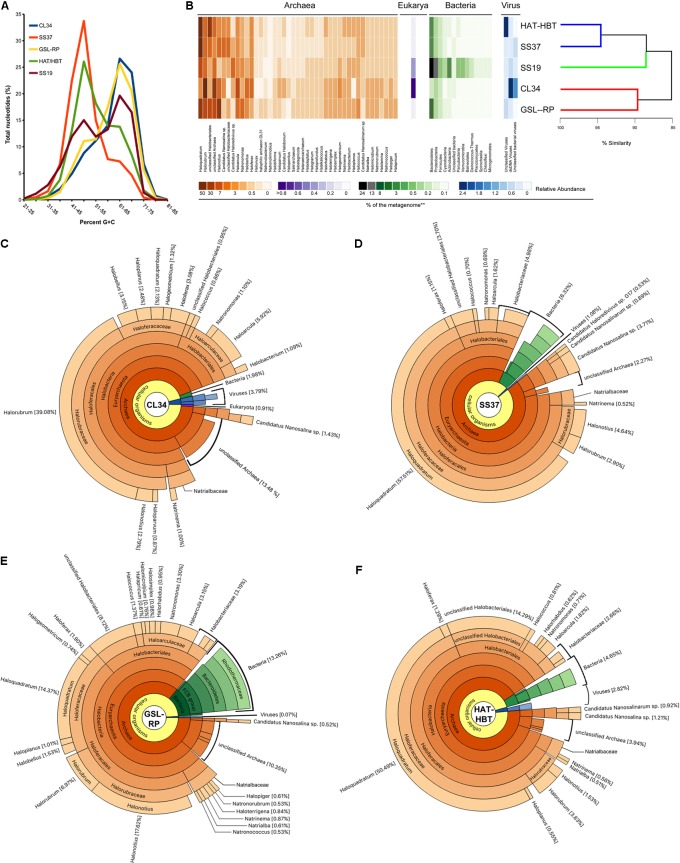
Metagenomic G+C content and taxonomic composition of aquatic salt-saturated environments. **(A)** G+C percentage of metagenomic reads, presented as percentage of total dataset in five unit interval bins, for Cáhuil Solar Saltern salt crystallizer pond (CL34), Santa Pola solar saltern 19% salt pond and salt crystallizer pond (SS19 and SS37, respectively), Great Salt Lake north arm Rozel Point (GSL-RP) and Lake Tyrrell January 2009 (HAT/HBT). **(B)** Heatmap of the scaled relative abundance of the main taxa of each salt-saturated metagenome. The taxa were scaled to zero mean and unit variance and clustered using the Euclidean distance and Ward linkage method to highlight patterns of diversity among samples. The dendrogram on the right represents the hierarchical clustering and similarity among metagenomes, and the colored lines highlight nodes that are statistically supported (SIMPROF). The highest taxonomic level assigned to the subset of metagenomic reads is shown for **(C)** CL34, **(D)** SS37, **(E)** GSL-RP, and **(F)** HAT/HBT. Only taxa with >0.5% relative abundance were graphed, except for reads assigned to Eukarya.

Since G+C content usually falls within a relatively narrow range of individual genomes and closely related taxa often have similar nucleotide compositions, the G+C composition of metagenomic reads can serve as a useful metric for comparing the overall community structure (Podell et al., 2014). The G+C profile of each salt-saturated metagenome exhibited one main peak, which was either at 51–55% G+C (SS37 and HAT/HBT) or 66–70% G+C (CL34 and GSL-RP; **Figure 2A**). However, the less saline SS19 sample exhibited two peaks within the same range as the G+C percentages of the salt-saturated metagenomes (**Figure 2A**). When clustering these metagenomes according to their taxonomic composition they grouped in agreement with their G+C content, CL34 and GSL-RP formed a distinct group (87.5% similarity; SIMPER) from the SS37, HAT/HBT and SS19 metagenomes (**Figure 2B**). The reads from all four salt-saturated metagenomes were assigned principally to the Archaea domain within the Halobacteriaceae family. However, CL34 was the only system in which *Halorubrum* was the most abundant genus (39.08%) and in which *Haloquadratum* was only a minor component of the community, accounting for just 2.13% of the total reads (**Figure 2C**). Conversely, in all other metagenomes, the genus *Haloquadratum* recruited the majority of reads (or second most in the case of GSL-RP): 14.54% in GSL-RP, 31.86% in HAT/HBT and 52.08% in SS37 (**Figures 2B,D–F**). Phylogenetic inference based on the 119 archaeal SSU rRNA gene fragments from the assembled metagenomic data set showed that there were two distinct *Halorubrum* populations in the CL34 metagenome (**Supplementary Figure 4A**).

The sum of sequences assigned to Eukarya, Bacteria, and Viruses comprised less than 14% of these aquatic salt-saturated metagenomes (**Figures 2C–F** and **Supplementary Data Sheet 2**). The majority of these non-archaeal reads were associated with bacterial taxa from the phyla Bacteroidetes, Proteobacteria (class Gammaproteobacteria) and Firmicutes (class Bacilli) (**Figures 2B–F** and **Supplementary Figures 4**, **5**).

Despite broad taxonomic similarities among the salt-saturated metagenomes analyzed, CL34 stood out from other samples not only in having *Halorubrum* as the dominant genus but also due to the low representation of *Haloquadratum*, which is the most dominant group of Halobacteriaceae in the other metagenomes (**Figure 2**). Although the dominance of *Haloquadratum* is common among microbial communities from salt-saturated aquatic environments (Oren, 2002, 2013a; Ghai et al., 2011; Ventosa et al., 2014), cases in which *Halorubrum* comprise the most abundant members of the microbial community have been described in other solar saltern crystallizer ponds (Pašic et al., 2005). The presence of *Haloquadratum* has been positively correlated with high Mg^2+^ concentrations in Lake Tyrrell (where Mg^2+^ concentrations reach up to 941 mM in summer; Podell et al., 2014). This is likely also the reason for the dominance of *Haloquadratum* in metagenomes from the Santa Pola saltern ponds SS33 and SS37 (Fernández et al., 2014) where the Mg^2+^ concentrations can rise to ∼2050 mM (**Table 2**). However, the correlation between high Mg^2+^ concentrations and the dominance of *Haloquadratum* seems not to be a generalized observation (see **Table 2**). (i) CL34 has nearly 15-fold fewer metagenomic reads assigned to *Haloquadratum* than Lake Tyrell during summer despite having a similar Mg^2+^ concentration of 924 mM (Podell et al., 2014); (ii) *Haloquadratum* dominates the solar saltern crystallizer ponds of Guerrero Negro 12 (Dillon et al., 2013) and Tunisian Sfax S5 (Trigui et al., 2011), although their Mg^2+^ concentrations are lower than in CL34; finally, (iii) both SSU rRNA V6 region Pyro-tag sequencing and metagenomic fosmid libraries revealed that *Haloquadratum* did not represent a significant component of the Dead Sea microbial communities during 1992 nor in 2007, despite Mg^2+^ concentrations up to 1675 mM (Bodaker et al., 2009). Thus, the concentration of Mg^2+^ alone does not explain the prevalence of *Haloquadratum* in salt-saturated aquatic environments.

**Table 2:** Ion concentrations of hypersaline environments included in this study (in mM). For Santa Pola SS37 concentrations were calculated considering Mg^2+^ was up to 5% (w/v) (Rodriguez-Valera et al., 1985), Lake Tyrrell values are those of the 5th of January 2009 (Podell et al., 2014), Great Salt Lake values are an average of the North Arm (Great Salt Lake N-A; Baxter et al., 2005), data for Guerrero Negro Exportadora de Sal pond 12 (Guerrero Negro 12) was obtained from Dillon et al. (2013), Tunisian saltern values corresponded to those of pool “S5” (Baati et al., 2008), Dead Sea ′07 concentrations correspond to the average composition during 2007 (Bodaker et al., 2009), and Slovenian Secovlje saltern correspond to those of a 28% salinity pond during August the 5th, 1997 (Gunde-Cimerman et al., 2000). N.A. = Not analyzed.

Site/Ion (mM)	Santa Pola SS37	Lake Tyrrell	Cáhuil saltern CL34	Great Salt Lake N-A	Guerrero Negro 12	Tunisian saltern S5	Dead Sea ′07	Slovenian Secovlje saltern
**Na^+^**	N.A.	2908	1798.3	4393.25	3682.07	4001.77	1705	4132.27
**K^+^**	N.A.	65	120.11	176.48	6.99	0.75	187	122.77
**Mg^+2^**	∼ 2050	941	923.72	349.72	706.03	477.27	1675	773.5
**Ca^+2^**	N.A.	5	5.44	6.99	4763.89	4090.27	422	N.A.
**Cl^-^**	N.A.	4100	N.A.	4936.11	142.85	400.27	5991	5190.41
**SO_4_^2-^**	N.A.	276.7	N.A.	226.62	254.36	374.77	281	271.71


The hypersaline lakes analyzed here are disturbed at a seasonal timescale (Baxter et al., 2005; Podell et al., 2014), while the salt concentrations of the Santa Pola Bras del Port, Guerrero Negro and Tunisian Sfax saltern ponds are maintained approximately constant by means of an artificially regulated flow regime (Trigui et al., 2011; Dillon et al., 2013; Ventosa et al., 2014). In the Cáhuil Solar Saltern, the initial water batch completes its precipitation cycles in no more than three weeks before halite is harvested, and the artisanal workers usually do not use the same ponds during each cycle. As proposed by Pašic et al. (2005), the shorter doubling-time of *Halorubrum* cultures (3.03 and 22.3 h at 45°C and 23°C, respectively) (Robinson et al., 2005) compared to *Haloquadratum* (1.5 to 5.5 days at 37°C) (Burns et al., 2004), could make *Haloquadratum* more affected by the frequency of disturbances of its environment. Thus, more disturbed salt-saturated aquatic environments (e.g., the Cáhuil and Slovenian Secovlje crystallizer ponds) are likely to be dominated by fast-growing groups, despite the presence of sub-optimal concentrations of Mg^2+^ (>700 mM; **Table 2**) (Gunde-Cimerman et al., 2000; Pašic et al., 2005). In contrast, more stable ecosystems are likely to be dominated by *Haloquadratum*, especially when Mg^2+^ concentrations are high.

### Metabolic Signatures of Microbial Communities From Hypersaline Environments

**FIGURE 3:**
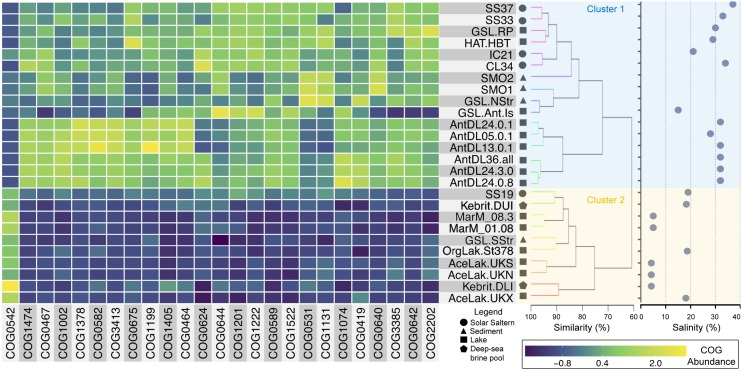
Functional composition of saline microbial communities. Dendrogram on the right shows the hierarchical clustering among the metagenomes. Clustering was done based on the abundance of COG functions among unassembled metagenomic sequences and normalized to the total number of sequences within an assigned COG function (**Supplementary Data Sheet 2**). The taxa were scaled to zero mean and unit variance and clustered using the Euclidean distance and Ward linkage method. Colored lines highlight nodes statistically supported (SIMPROF). Metagenome name abbreviations, salinity, references, and further information are detailed in **Supplementary Data Sheet 2**. Blue and yellow backgrounds highlight metagenomes from salt-saturated and sub-saturated hypersaline environments, respectively. Heatmap represents the relative abundance of COGs that showed significant differences between the sub-saturated and salt-saturated clades (**Supplementary Data Sheet 2**). COGs were ranked based on the adjusted *p*-value obtained from multiples paired *t*-tests with a *p* < 0.05 as cutoff.

The functional signature of hypersaline microbial communities was assessed here by comparing the abundance of all COG functions assignable to the reads of their metagenomes. The COG abundance profiles of the final 26 metagenomes clustered into two groups (PERMANOVA *p*-value < 0.001). These were comprised principally of microbial communities from either salt-saturated (>30% salinity) or sub-saturated (<30% salinity) hypersaline environments, respectively (**Figure 3**). The only exceptions were the GSL-Ant-Is and IC21 metagenomes, which clustered with the salt-saturated metagenomes despite having 15 and 21% salinity, respectively (**Figure 3**). In agreement with their highly similar microbial community compositions (Moller and Liang, 2017), the IC21 metagenome displayed the highest similarity of functional genes abundance to CL34 (**Figure 3**). The analysis presented here showed that the functional category “Replication, recombination, and repair” presented the most COGs (19) among the 100 most abundant functions in all the hypersaline metagenomes assessed here (**Supplementary Data Sheet 2**). The main functions of these overrepresented categories were those involved in nucleotide excision repair (COG0178, COG1199, and COG1061), site-specific recombination (COG0468, COG0582, and COG4974), helicase activity (COG0210, COG0553, and COG1201), replication (COG0188, COG0417, COG1241, and COG1474) and DNA methylases of restriction-methylation systems (COG0270, COG0863, COG0827, COG1002, and COG1403).

Regarding COGs involved in nucleotide excision repair, the Uvr protein complex (UvrABCD/COG0178) of Bacteria and Archaea repairs UV-induced DNA lesions (Sancar and Rupp, 1983; Berquist et al., 2005). Specifically, archaeal COG0433 have been linked to DNA helicase activity for loading and subsequent unwinding of the DNA duplex (Constantinesco et al., 2004). Similarly, genes associated to COG0433 and COG4974 correspond to archaeal homologs of FtsK (Iyer et al., 2004) and XerD (Iyer et al., 2004), respectively, acting in concert to resolve dimeric circles and the consequent segregation of daughter chromosomes during cell division (Iyer et al., 2004). The high levels of solar radiation received by microorganisms from hypersaline environments would likely favor the presence of the Uvr systems to repair the UV radiation-induced damage inflicted to their DNA.

Type II (COG1002) and type IV (COG1403) methylases were also found to be overrepresented among these hypersaline metagenomes, suggesting that these enzymes may alleviate the negative effects that high salt concentrations impose on DNA and RNA structure and stability. Methylases work with restriction endonucleases, forming restriction-methylation systems, to protect the host from foreign DNA (Madhusoodanan and Rao, 2010). Correspondingly, some of these overrepresented helicases and methylases (COG0553, COG1002 COG1201, COG0863, COG1061, and COG1403) have been linked to genomic loci rich in anti-viral defense mechanisms in Archaea and Bacteria (Makarova et al., 2011). Thus, the high proportion of sequences associated to these functions, might be linked to the elevated levels of genetic exchange described in genomes of both halophilic archaea (Nelson-Sathi et al., 2012; DeMaere et al., 2013; Podell et al., 2013; Becker et al., 2014) and bacteria (e.g., *Salinibacter ruber*; Mongodin et al., 2005).

### Functional Genomic Signatures of Salt-Saturated Versus Sub-Saturated Hypersaline Microbial Communities

The abundance of 25 of the 100 most abundant COGs functionally distinguished salt-saturated from sub-saturated hypersaline metagenomes (**Figure 3** and **Supplementary Data Sheet 2**). Of these 25 differentially abundant COGs, salt-saturated metagenomes had a higher abundance of sequences within the categories “Mobilome: prophages, transposons,” “Replication, recombination and repair,” “Transcription,” “Amino acid transport and metabolism” and “Signal transduction mechanisms.” In contrast, sub-saturated metagenomes only displayed significantly higher percentages of sequences associated with the category “Post-translational modification, protein turnover, and chaperones” (COG0542).

Sequences associated to the COG category “Mobilome: prophages, transposons” included those responsible for DNA mobilization and retroviral integration (COG0675, COG0582, and COG3385). Their higher representation among salt-saturated hypersaline metagenomes can be attributed to the high abundance of viruses in these environments. Virus counts appear to be higher in crystallizer ponds than in sub-saturated hypersaline waters (10^9^ vs. 10^7^ viruses per mL respectively) (Santos et al., 2012; Moller and Liang, 2017). Also, three main transcriptional regulator families were overrepresented in salt-saturated environments: the ArsR (COG0640); Lrp (COG1522); and TrmB (COG1378). The ArsR members regulate the expression of operons linked to stress-inducing concentrations of di- and multivalent heavy metal ions, but also can regulate oxidative stress response in Archaea (Karr et al., 2017). As shown in *Halobacterium salinarum*, Lrp proteins regulate many genes involved in amino acid synthesis, central carbon metabolism, transport processes and transcription regulation (Peeters et al., 2015). Finally, TrmB-like proteins are involved in the regulation of sugar metabolism in response to the direct binding of sugar ligands, but also have an essential role in chromatin organization (Peeters and Charlier, 2010). Overall, these characteristics point toward the presence of dynamic genomes in salt-saturated hypersaline environments, with a high representation of gene functions involved in the integration of mobile DNA elements and stress tolerance.

Most of the other functional features distinguishing salt-saturated from sub-saturated microbial communities are likely due to the overwhelming dominance of “high-salt-in” halophiles in the former (i.e., principally Archaea from the Halobacteriaceae family and Bacteria of the genus *Salinibacter*) (Oren, 2006a,b). Thus, the physiological differences between the highly specialized “high-salt-in” microorganisms, compared to the metabolically and taxonomically more diverse halophiles with “low-salt-in” strategies (Oren, 2013a), are likely determining various functional differences observed among the hypersaline metagenomes analyzed here.

## Conclusion

The Cáhuil Solar Saltern presents five ponds with different salinity, pH, and temperature, inhabited by different microbial communities presenting distinctive pigments, light scattering properties, and DNA content. Metagenome sequences of the salt crystallizer pond of this artisanal solar saltern are surprisingly dominated by sequences associated with the genus *Halorubrum*, with a considerably lower representation of *Haloquadratum*, generally described as the dominant halophilic archaeal genus of these ecosystems. Future studies are required to understand the factors and mechanisms modulating the capacity of *Halorubrum, Haloquadratum*, and other Halobacteriaceae to dominate these extreme environments.

The functional genomic comparison performed here revealed that hypersaline microbial metagenomes include a high proportion of predicted protein functions related to nucleotide excision repair, helicases, replication and restriction-methylation systems. Additionally, our results show the existence of a distinctive COG profile signature differentiating between microbial communities from salt-saturated and sub-saturated hypersaline environments, mainly related to mobile elements and stress response.

Overall, this study provides a detailed characterization of the crystallizer pond from the Cáhuil Solar saltern. This study is the first to describe a distinctive signature regarding the functional repertoires of salt-saturated and sub-saturated hypersaline environments. Further studies allocating more replicates and higher metagenomic coverage of this and other salt production systems will allow a better understanding of the drivers determining functional adaptations to extreme hypersaline habitats.

## Author Contributions

AP, ND, CH-C, RDI, EA, and NT conceived and designed the study. AP, CH-C, ND, JS, and KH collected the field samples. CH-C and GE performed the FCM analyses. JS performed the pigment analyses. AP, EA, SP, JU, and NT designed the sequencing approach and bioinformatics analyses. CH-C and ND extracted nucleic acids and performed fingerprinting analyses. AP and ND conducted the chemical measurements. AP, CH-C, ND, SP, SR-F, and RDI analyzed the data. RDI, EA, OU, and NT contributed with data or analytical tools. AP and CH-C wrote the paper with contributions from all authors. All authors revised and approved the final version of the manuscript.

## Conflict of Interest Statement

The authors declare that the research was conducted in the absence of any commercial or financial relationships that could be construed as a potential conflict of interest.

## Footnotes

1 http://genome.jgi-psf.org/genome-projects/

2 www.geneious.com

3 http://bosque.udec.cl/software/tundra.html
