# Supplementary material for: Distinctive Archaeal Composition of an Artisanal Crystallizer Pond and Functional Insights Into Salt-Saturated Hypersaline Environment Adaptation
Source: Front Microbiol. 2018 Aug 14;9:1800. doi: 10.3389/fmicb.2018.01800 (PMC6102401; doi:10.3389/fmicb.2018.01800)
Supplement: Supplementary file 1 [file Data_Sheet_1.doc]

*Supplementary Material*

Distinctive archaeal composition of an artisanal crystallizer pond and functional insights into salt-saturated hypersaline environment adaptation

Alvaro M. Plominsky, Carlos Henríquez-Castillo, Nathalie Delherbe, Sheila Podell, Salvador Ramírez-Flandes, Juan A. Ugalde, Juan Francisco Santibañez, Gerit van der Engh, Kurt Hanselmann, Osvaldo Ulloa, Rodrigo De la Iglesia, Eric E. Allen*, Nicole Trefault*

*** Correspondence:** Nicole Trefault ([nicole.trefault@umayor.cl](mailto:nicole.trefault@umayor.cl)), Eric E. Allen: [eallen@ucsd.edu](mailto:eallen@ucsd.edu)


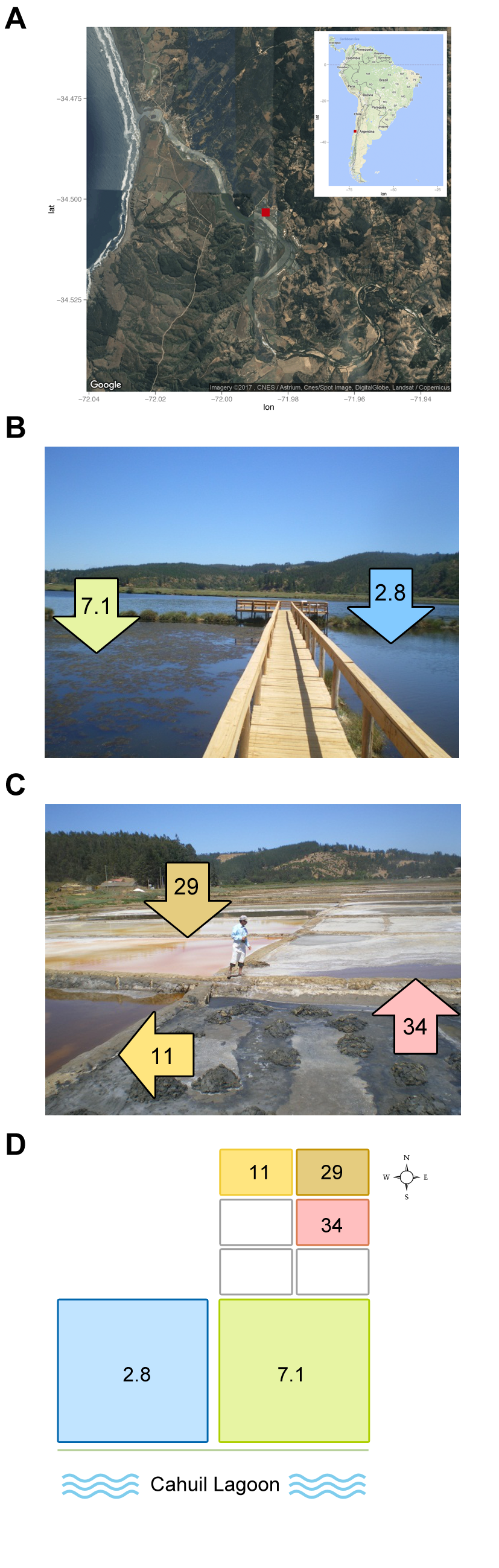
**1 Figures and Tables**

**Figure S1.** The geographical location of the Cáhuil Solar Saltern and the ponds analyzed in this study. **(A)** The solar saltern, located at the North shore of the Cáhuil Lagoon, is signaled with a red square. The location of Cáhuil is also indicated with a red square in South America map, at the upper right. **(B)** Photograph of the Cáhuil Lagoon (further back), and the first two solar saltern ponds. **(C)** A photograph of the three last saltern ponds of the salt harvesting process. The numbers within the colored arrows indicate the total salt concentration (% [w/v]) of each pond. **(D)** The scheme shows the distribution of the solar saltern ponds during sample collection. Colored squares correspond to the ponds analyzed in this study, and the numbers within them indicate the total salt concentration (% [w/v]) of each pond. Hollow squares correspond to ponds of a parallel salt-harvesting process that were not analyzed in this study.


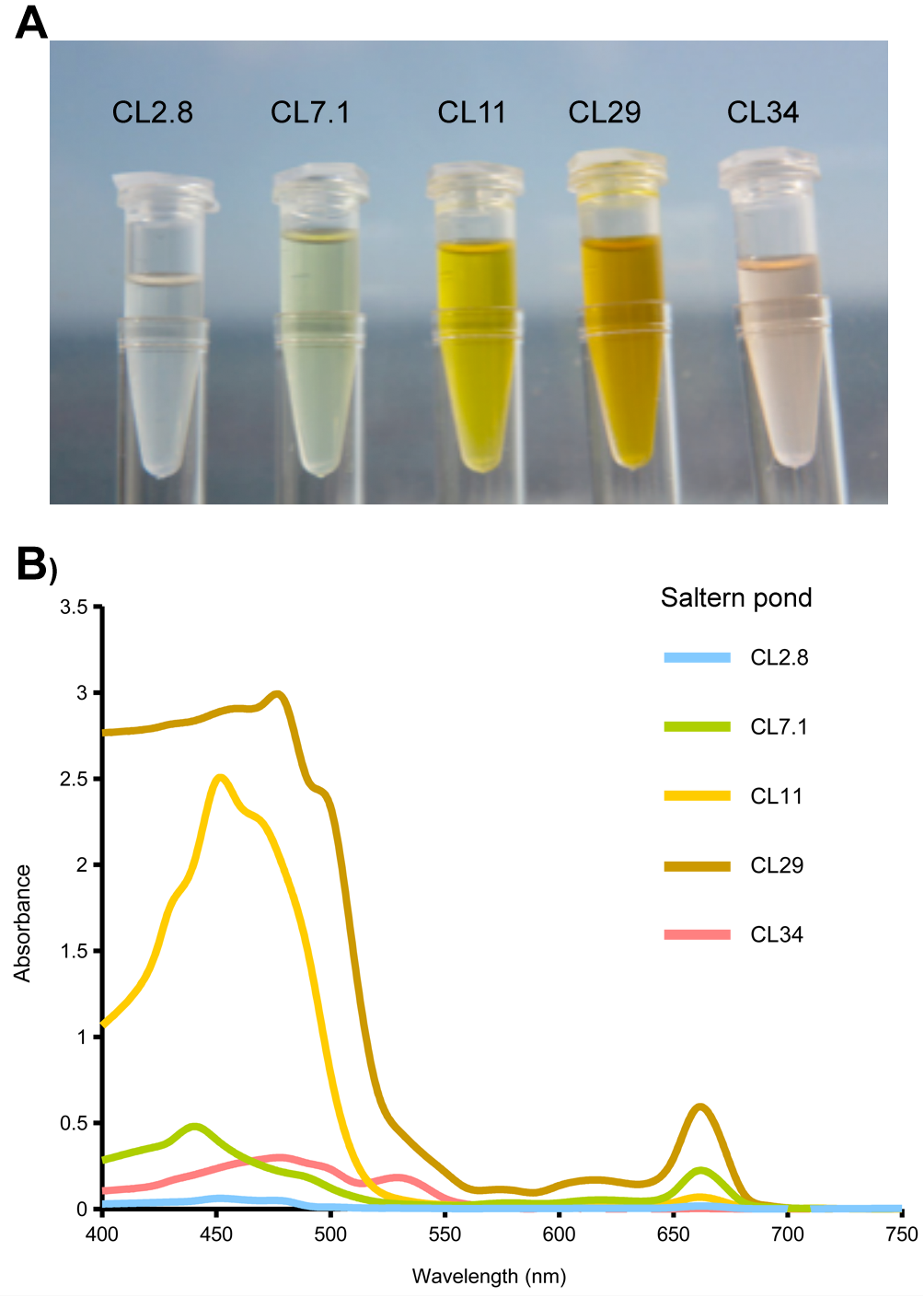


**Figure S2.** Pigments of the Cáhuil Solar Saltern. **(A)** A photograph of the pigments extracted from each pond. **(B)** Absorption spectra of total pigments extracted from the microbial communities of each pond.


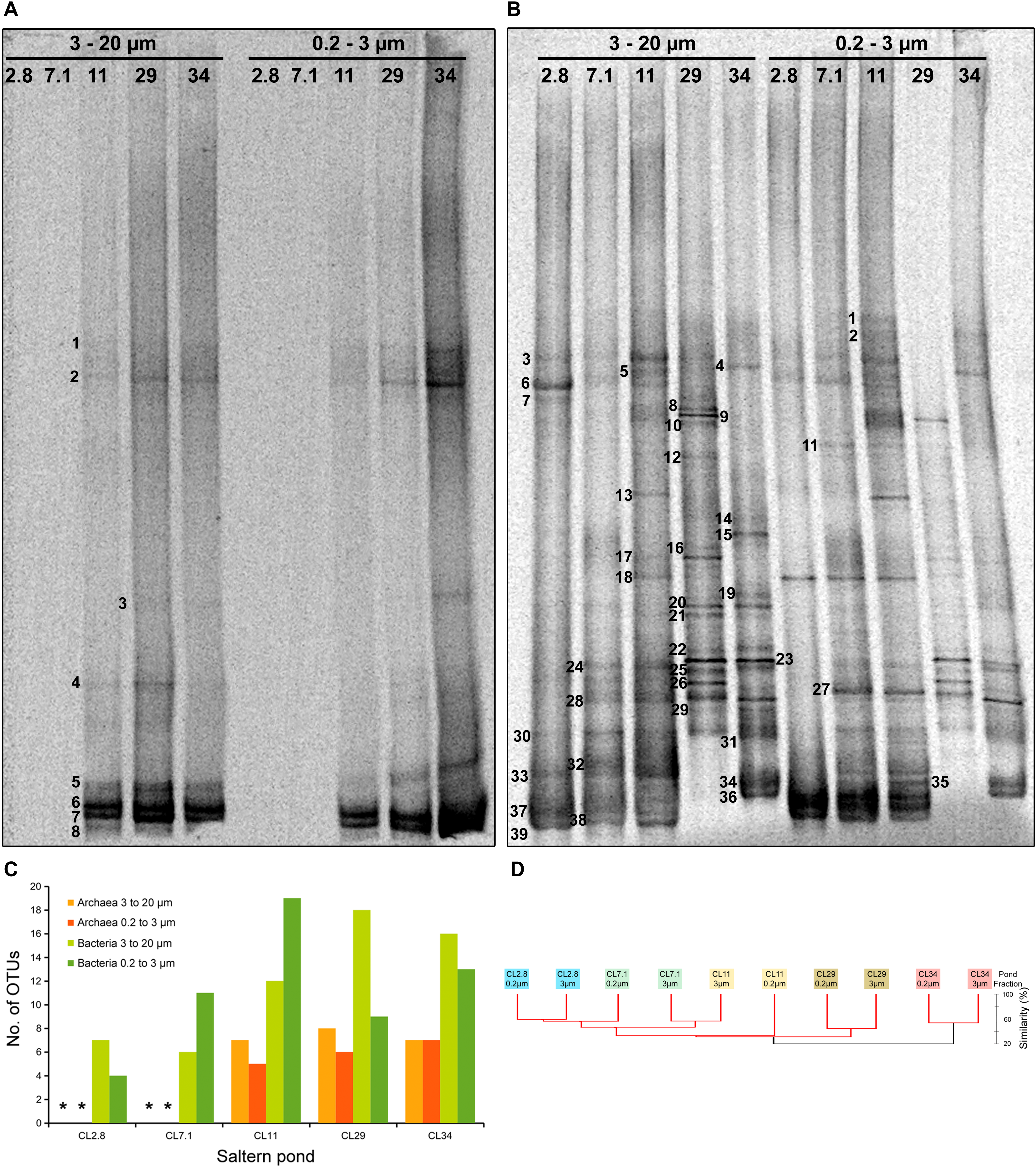


**Figure S3.** Negative images of DGGE gels containing **(A)** Archaea and **(B)** Bacteria PCR-amplified fragments of the community SSU-rRNA genes from the 3 to 20 µm and 0.2 to 3 µm size fractions of each pond. All the bands considered in the richness and community clustering analysis (**Figure 1D**) are enumerated at the left side of the corresponding band (only the first band of each Operational Taxonomic Units [OTU], from up to down and from left to right, was enumerated). **(C)** Number of Archaeal and Bacterial OTUs detected in the 3 to 20 µm and 0.2 to 3 µm size fractions of each pond. The '*' denote that no Archaeal amplicons were detected in the PCR of these samples. **(D)** Hierarchical clustering of the 3 to 20 µm and 0.2 to 3 µm size fractions of each pond, according to the presence/absence of bacterial OTUs (Bray-Curtis similarity and the furthest-neighbor algorithm). Statistically supported nodes (SIMPROF) are denoted with red lines.


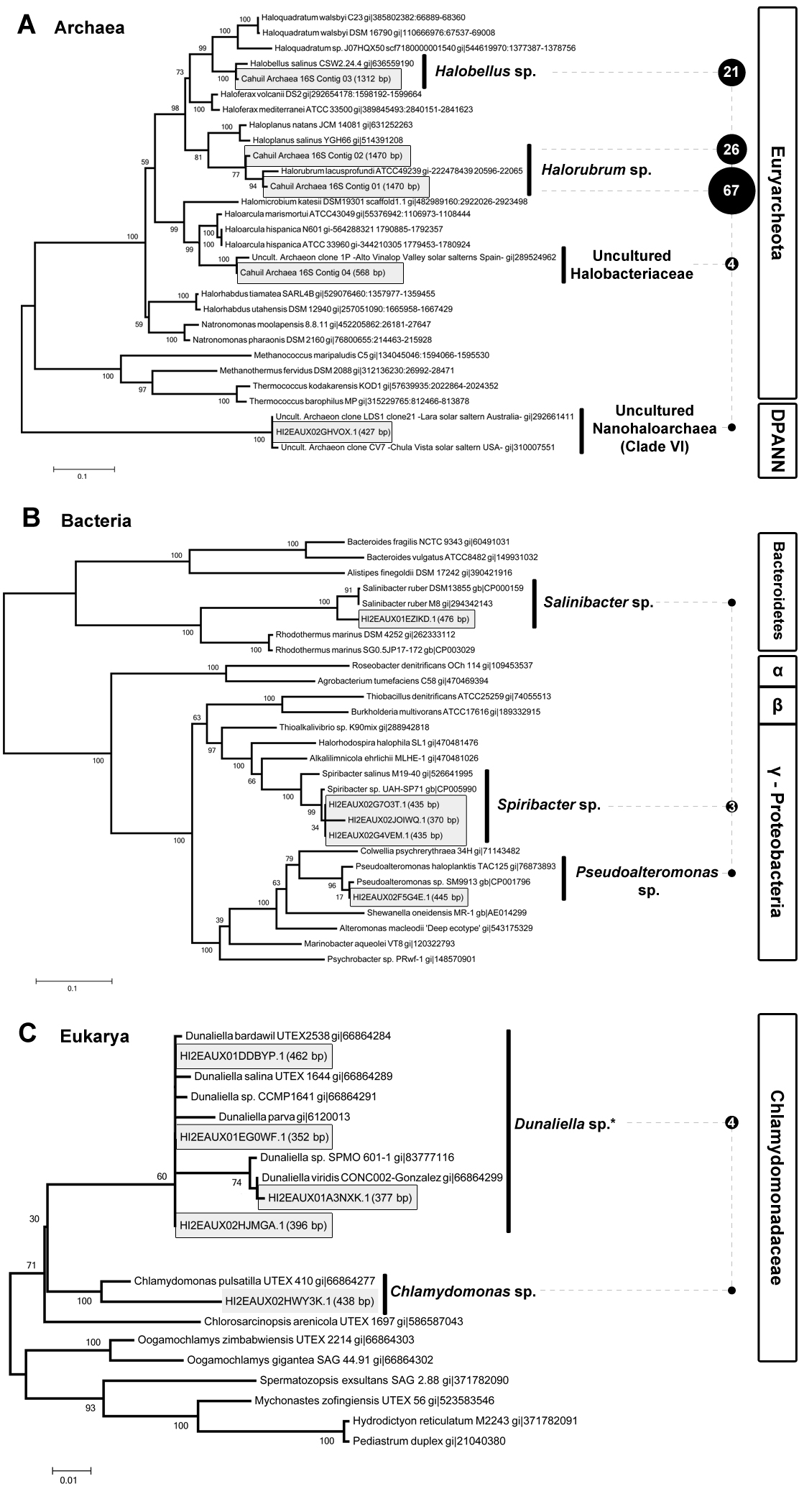


**Figure S4.** Phylogenetic trees of rRNA gene fragments from CL34 metagenome. (A) Archaeal SSU-rRNA. (B) Bacterial and (C) eukaryotic LSU-rRNA phylogenetic trees. Numbers at nodes represent bootstrap values and scale bars show an average number of substitutions per site. Contigs and singleton sequences from CL34 are highlighted inside gray squares. The total sequence length of the contigs and singletons are shown in parenthesis, and the reads assembled by each contig are denoted inside a black circle whose size is proportional to their number (singletons do not have a number inside the circle). '*' denotes that there were also reads assigned to Dunaliella sp. SSU-rRNA gene from chloroplasts and mitochondria (Supplementary Figure S5).


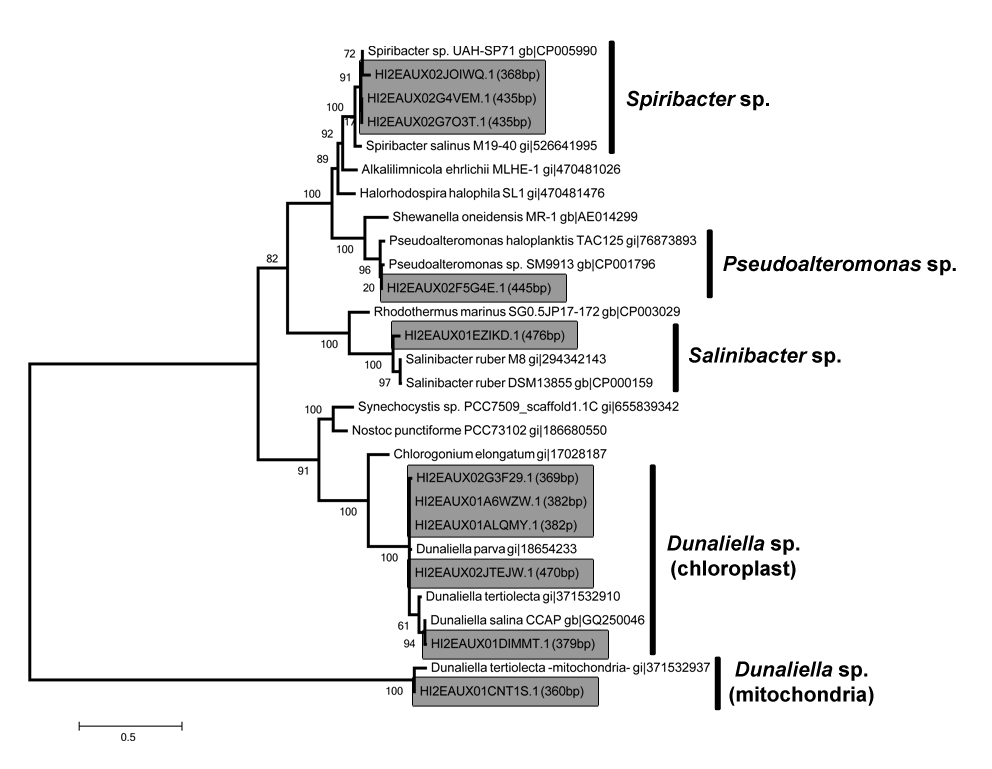


**Figure S5.** Bacterial SSU-rRNA Maximum Likelihood phylogenetic tree including *Dunaliella* sp. chloroplast and mitochondria SSU-rRNA gene fragments from CL34 metagenome. Numbers at nodes represent bootstrap values and scale bar shows the average number of substitutions per site. Sequences from CL34 are highlighted inside grey squares.
